# Screening and phylogenetic characterization of tick-borne pathogens in a population of dogs and associated ticks in Egypt

**DOI:** 10.1186/s13071-022-05348-x

**Published:** 2022-06-21

**Authors:** Asmaa A. Hegab, Hussein M. Omar, Mai Abuowarda, Souzan G. Ghattas, Nisreen E. Mahmoud, Magdy M. Fahmy

**Affiliations:** 1 Department of Parasitology, Animal Health Research Institute, Agriculture Research Center, Dokki, Egypt; 2 Department of Parasitology, Faculty of Veterinary Medicine, Cairo University, Giza square, PO Box 12211, Giza, Egypt

**Keywords:** *Anaplasma*, *Babesia*, *Ehrlichia*, Egypt, *Hepatozoon*, PCR, *Rhipicephalus sanguineus* (s.l.), Tick-borne diseases

## Abstract

**Background:**

The incidence or recurrence of tick-borne diseases (TBDs) in animals and humans is increasing rapidly worldwide, but there is insufficient information about TBDs infecting dogs in Egypt. Thus, the present study was conducted to screen and genetically identify tick-borne pathogens (TBPs) in dogs and associated ticks by microscopic examination and polymerase chain reaction (PCR).

**Methods:**

In Cairo and Giza governorates, 208 blood samples were collected from dogs of different breeds, ages, and sex. In addition, 1266 dog-associated ticks were collected (546 ticks were used to prepare hemolymph smears, and 720 ticks were kept in 70% ethanol until PCR analysis). PCR was applied to 124 dog blood samples and 144 tick pools prepared from 720 ticks.

**Results:**

All ticks collected from dogs were *Rhipicephalus sanguineus* (s.l.). Microscopic examination revealed that TBP prevalence among dogs was 23.56% (49/208), including *Anaplasma* and *Ehrlichia* with 11.1% (23/208) and *Babesia canis* with 8.2% (17/208). *Hepatozoon canis* was not detected in blood smears. Co-infections with two pathogens were visible in 4.33% (9/208) of examined dogs. The prevalence of TBPs in hemolymph smears was 45.97% (251/546) including 35.89% (196/546) for *H*. *canis*, 8.1% (44/546) for *B. canis*, and 2.01% (11/546) for *Anaplasmataceae* (*A. phagocytophilum*, *A. marginale, A. platys*, and *E. canis*). The overall molecular prevalence rate of TBPs was 25.81% and 29.17% in the blood of examined dogs and in ticks, respectively. The molecular prevalence of Anaplasmataceae family, *Babesia canis*, and *H*. *canis* in dog blood samples was 19.35%, 6.45%, and 0.0%, respectively, while in ticks, it was 20.83%, 5.55%, and 2.8%, respectively. A sequential analysis identified six different species of TBPs, namely *B. canis vogeli, Hepatozoon canis, A. phagocytophilum*, *A. marginale, A. platys*, and *E. canis.* The obtained sequences were submitted to GenBank and assigned accession numbers.

**Conclusions:**

The present study detected a wide range of TBPs (*B. canis, H. canis, A. platys, A. phagocytophilum, A. marginale*, and *E. canis*) that are considered a threat to domestic animals and humans in Egypt. *Hepatozoon canis* and *A. marginale* were reported in dogs and associated ticks for the first time in Egypt.

**Graphical Abstract:**

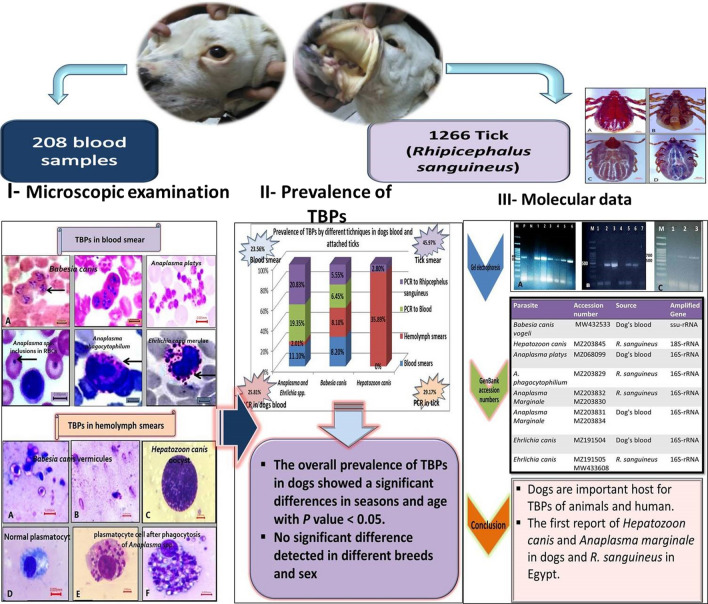

## Highlights

- Traditional and molecular techniques were used to screen domestic dogs and attached ticks for TBPs.
- A wide range of TBPs in dogs and attached ticks was detected in Egypt.
- Some accidental pathogens, such as *A. marginale* from other hosts, were identified in dogs.
- Some canine isolates from Anaplasmataceae family have high homology to those isolated from humans.

## Background

Ticks, as blood-feeding ectoparasites, directly damage vertebrate hosts during blood sucking and indirectly by transmitting many pathogens such as bacteria, viruses, and protozoa [1–4]. *Rhipicephalus sanguineus* (s.l.) is a widespread tick responsible for transmitting many pathogens, such as *Babesia canis vogeli*, *Ehrlichia canis*, and *Anaplasma platys* to dogs. Furthermore, many authors recorded fatal human cases due to *Ehrlichia* and *Anaplasma* spp. infection [5, 6]. Tick-borne pathogens (TBPs) are the leading cause of morbidity and mortality in pet dogs and a major public health concern. To date, three protozoa (*Babesia*, *Theileria,* and *Hepatozoon*) and five bacterial genera (*Anaplasma*, *Ehrlichia*, *Rickettsia*, *Coxiella*, and *Bartonella*) are recorded in dogs globally [7]. Dogs subclinically infected with TBPs act as reservoirs of these diseases for their owners and contact animals [8, 9]. Signs of TBDs are usually diffuse and overlapping, particularly in mixed infections; therefore, accurate diagnostic methods are required for a correct treatment and control strategy [10]. TBP diagnoses are usually based on microscopic examination of blood smears. Although these morphology-based methods save time and money, they are less sensitive and need highly skilled investigators. Thus, molecular techniques confirm the identity of canine blood parasites [11]. The molecular methods have higher sensitivities and specificities in detecting TBPs in peripheral blood and tick tissues than traditional techniques [12, 13]. Additionally, molecular technique result analysis helped better understand new species that infect dogs. For example, some authors genetically divided dog piroplasms into three groups: true *Babesia*, *B. microti*, and *B. conradae* group [14]. As pet dog ownership has increased recently in Egypt and constitutes a potential TBD reservoir, human infections will increase. Therefore, the current study was conducted to detect and genetically identify TBPs in dogs (household and kenneled) and associated ticks. A combination of screening microscopic examination and molecular techniques [polymerase chain reaction (PCR) and sequencing] were applied to address these issues.

## Methods

### Sample collection

Blood samples were collected from 144 tick-infested dogs and 64 previously infested dogs (both sexes, different ages, and breeds) from government and private clinics and shelters in Cairo and Giza governorates (*n* = 208) based on sample size calculation using Open EPI free software https://www.openepi.com/SampleSize/SSPropor.htm and a confidence level of 95%. Ticks were collected directly from animals, kept in well-ventilated jars, and transported to the laboratory. From 1266 engorged ticks, 546 were used to prepare hemolymph smears, and 720 ticks were stored in 70% alcohol at − 20 °C till DNA extraction. Morphological identification of ticks was performed microscopically using identification keys [15]. Blood samples were collected from the cephalic vein into 5-ml EDTA tubes and then divided into two parts; one part was stored at − 20 °C till DNA extraction. The other part was used to prepare thin blood smears according to the method described by [16]. Smears were fixed by absolute methyl alcohol and stained with 10% Giemsa solution. The stained slides were examined with an oil immersion lens (1000 ×). The TBP stages in smears were measured according to the method described by [17].

### Hemolymph smear preparation

Five hundred forty-six ticks were washed twice with 70% ethanol and once with phosphate-buffered saline before preparing hemolymph smears, as [18] described.

### DNA extraction

One hundred twenty-four blood samples were analyzed by PCR based on using the Open EPI free software https://www.openepi.com/SampleSize/SSPropor.htm.htm and a confidence level of 95%. This study used 720 ticks to prepare 144 tick pools by crushing 5 ticks collected from each dog as a pool before DNA extraction. DNA extraction from tick and blood samples used blood and tissue extraction kits (G-spin total DNA extraction kit from Intron Biotechnology-Korea) following the kit manufacturer's instructions.

PCR reactions were performed in a total volume of 50 μl using 25 µl of Cosmo PCR red master mix (Willowfort Co.,UK) with 20 pmol of each primer (Table 1) and 5 µl of extracted DNA, completed with sterile nuclease-free water. Amplification was performed using a programmable conventional thermocycler (Biometra, Göttingen, Germany) according to the thermal profile (Table 2).

**Table 1:** Primers sequences used in the current study

Target organism	Primer sequence	Amplified gene/ length (bp)	Reference
*Babesia* spp*.*	5’-GTCTTGTAATTGGAATGATGG-3’ 5’-CCAAAGACTTTGATTTCTCTC-3’	ssu-rRNA/560	[12]
*Hepatozoon canis*	5’-ATACATGAGCAAAATCTCAAC-3’ 5’- CTTATTATTCCATGCTGCAG-3’	18S-rRNA/666	[19]
Anaplasmataceae	5’-TTTATCGCTATTAGATGAGCCTATG-3’ 5’-CTCTACACTAGGAATTCCGCTAT -3’	16S-rRNA/450	[20]

**Table 2:** Thermal profile used for PCR procedures

Parasite Step	*Babesia*		Cycle no.	*Hepatozoon canis*		Cycle NO	Anaplasmataceae		Cycle no.
	Temp	Time		Temp	Time		Temp	Time	
Initial denaturation	94 °C	5 min	1	95 °C	5 min	1	95 °C	5 min	1
Denaturation	94 °C	30 Sec	35	95 °C	30 Sec	35	95 °C	30 Sec	40
Annealing	49 °C	30 Sec		51 °C	30 Sec		55 °C	30 Sec	
Extension	72 °C	1 min		72 °C	1.5 min		72 °C	30 Sec	
Final extension	72 °C	10 min	1	72 °C	10 min	1	72 °C	10 min	1

The amplified products were electrophoresed on 1.5% agarose gels in 1× Tris Acetate EDTA (TAE). The agarose gel was stained with ethidium bromide and visualized by UV transillumination. The amplified fragment's size was compared to a 100-bp DNA molecular weight marker (Genedirex 100-bp DNA ladder H3 RTU). For each assay, the genomic DNA of known blood parasites was used as a positive control while nuclease-free water was used as a negative control [21].

### DNA sequencing and phylogenetic analysis

Positive PCR products of *Babesia* and *Hepatozoon canis* and *Anaplasma* species were excised from the gel and then purified using a QIAquick purification kit (Qiagen, Germany) according to the manufacturer’s protocol. Purified products were subjected to one-direction Sanger sequencing in the ABI 3500 Genetic Analyzer (Applied Biosystems, USA) using the Big Dye Terminator V3.1 kit (Applied Biosystems). After that, the PCR products’ nucleotide sequences were analyzed for similarity with those available in the GenBank using the BLAST server on the NCBI website. Obtained sequences were compared to sequences recorded in Egypt and some selected sequences isolated from dogs, ticks, and other animals worldwide. The analysis was done using the Clustal W, BioEdit software (ver. 7.0.9). A maximum likelihood method and Tamura-Nei model were constructed for the phylogenetic tree using Mega 6.06 software, and bootstrap analysis was obtained with 1000 replicates. A similarity matrix was carried out using the DNASTAR program (Lasergene, version 8.0). The genetic distance values of species variations were analyzed with the Meg-Align project of DNSTAR software [22].

### Nucleotide sequence accession numbers

A partial sequence of ssu-rRNA of *Babesia canis vogeli* gene, 18S-rRNA of *Hepatozoon canis*, and a partial sequence of 16S-rRNA of *A. platys, n. phagocytophilum*, *A. marginale*, and *E. canis* from the dog blood samples and *R. sanguineus* (s.l.) were submitted to GenBank, which assigned them the following accession numbers of pathogens and sequence length (*B. canis vogeli*: MW432533 (534 bp), *H. canis:*MZ203845 (600 bp), *A. platys:* MZ068099 (427 bp), *A. phagocytophilum:* MZ203829 (233 bp), *A. marginale:* MZ203830 (251 bp), MZ203832 (261 bp), MZ203831 (271 bp), MZ203834 (260 bp), *E. canis*: MW433608 (421 bp), MZ191505 (310 bp), and MZ191504 (350 bp).

## Statistical analysis

Data obtained from the blood smears were analyzed using SPSS. Pearson chi-square (χ^2^) tested the effect of different seasons, age, sex, and dog breeds on the prevalence of TBPs by blood smears. *P*-values < 0.05 were considered statistically significant [23].

## Results

### Tick identification

**Fig. 1:**
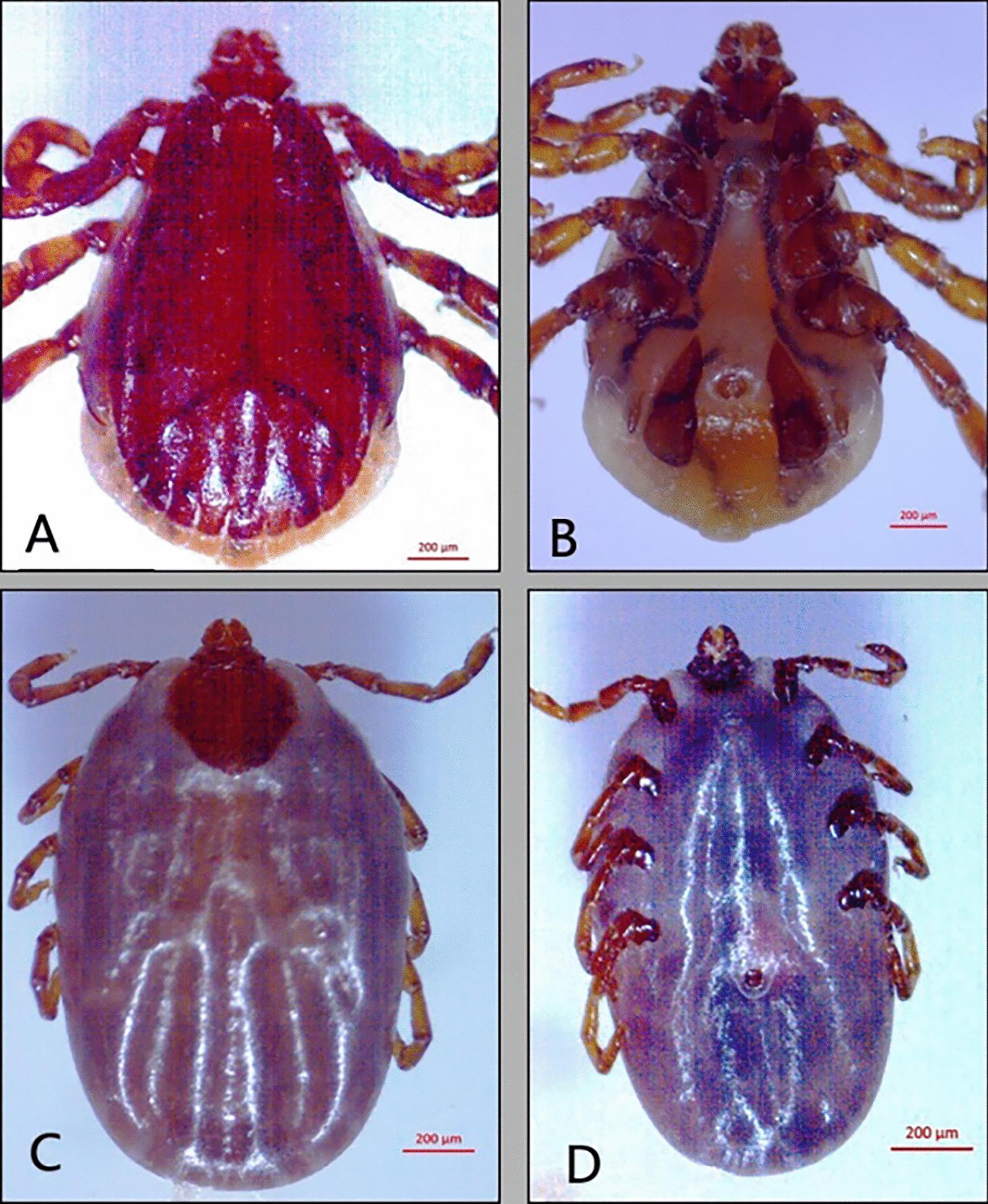
Male and female *R. sanguineus (s.l.)*: (**A**) Male dorsal view, (**B**) Male ventral view, (**C**) female dorsal view, (**D**) female ventral view (bare = 200 µm)

All ticks collected from dogs in the present study were morphologically identified as *R. sanguineus* (*s.l.*). They are small ticks that have an elongated body with reddish-brown coloration. They have hexagonal basis capituli with pointed lateral angles and short palps and eyes; 11 festoons are present, but they are usually inornate. Males have a triangular adenal plate, and females have a wide U-shaped genital opening. The tail of the spiracular plate in male and female ticks is narrower than the festoon (Fig. 1).

### Microscopic finding

**Fig. 2:**
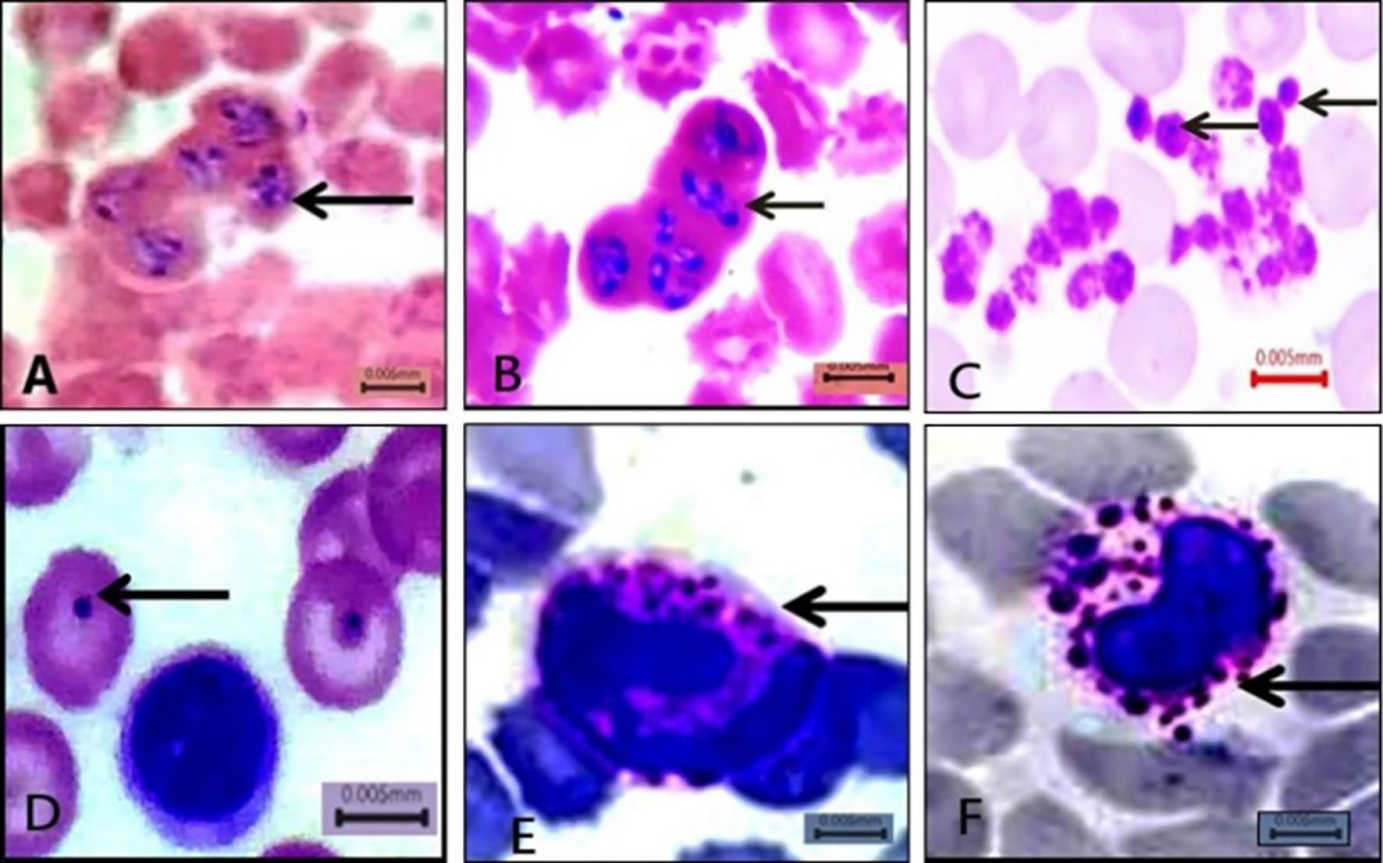
Giemsa-stained dog blood smears showing (**A** and **B**) *B. canis*, **C** *Anaplasma platys* inclusion inside thrombocytes, **D** *Anaplasma* inclusion inside RBCs. **E** *A. phagocytophilum* morulae inside neutrophil, **F** *E. canis* morulae inside monocyte (X1000 bare = 5 µm)

**Fig. 3:**
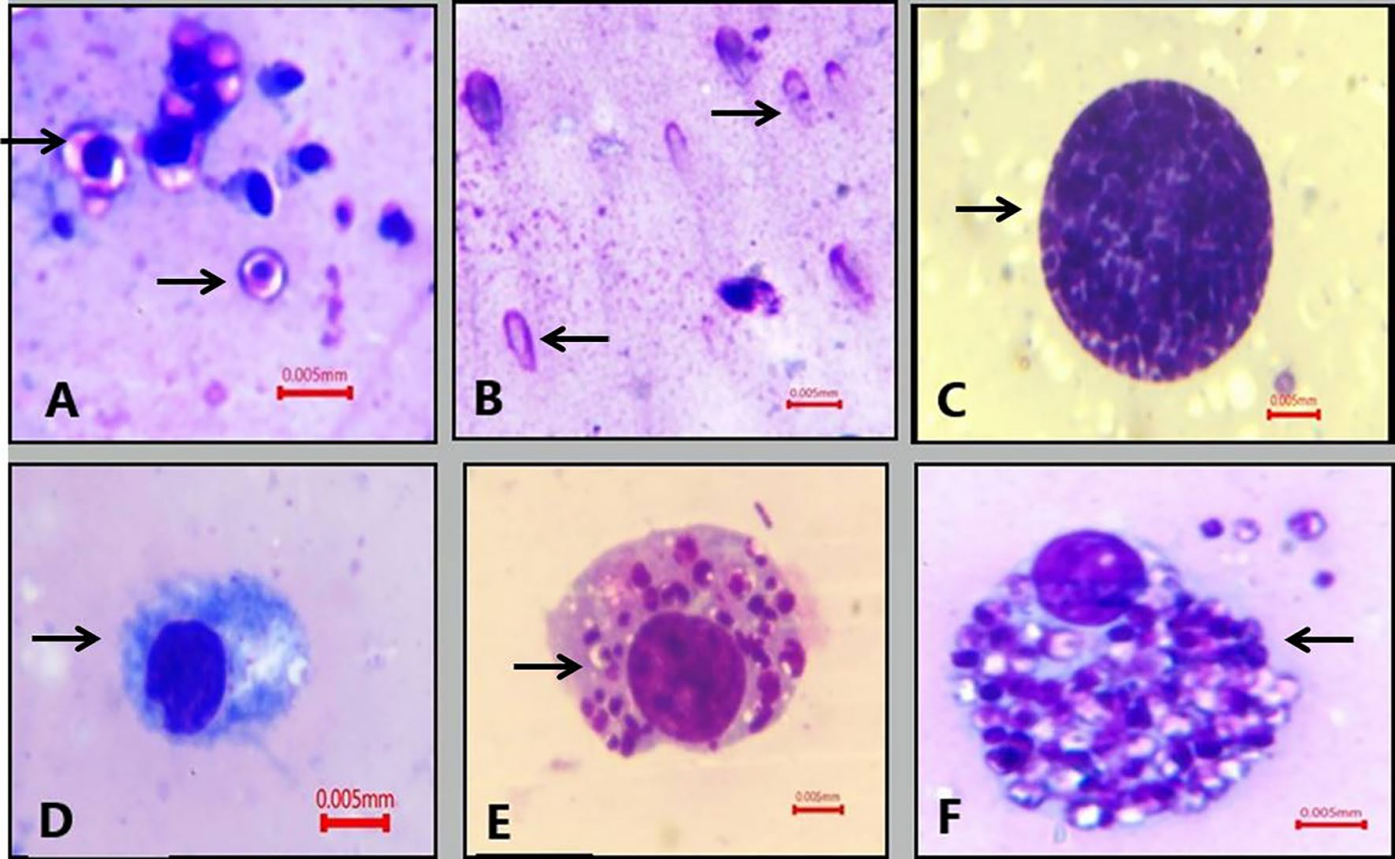
Giemsa-stained hemolymph smears of *R. sanguineus* (s.l.) showed **A**: spherical and amoeboid form of *Babesia canis*. **B**: Mature club-shaped vermicules of *B. canis*. **C**: *Hepatozoon canis* mature oocysts. **D**: Non-infected plasmatocyte cell. **E**: Plasmatocyte cell after phagocytosis of *Anaplasma* spp. **F**: Plasmatocyte eliminate *Anaplasma* spp. by nodulation (1000×, bare = 5 µm)

Giemsa-stained thin blood smear illustrated the presence of *B. canis* merozoites. They appeared polymorphic in shape, either typically large pear-shaped or oval. More than one piroplasm was detected in a single RBC, and the infected RBCs appeared clumped together, with sizes ranging from 3.9 to 4.8 µm. Regarding the Anaplasmataceae family in blood smears, *Anaplasma* inclusions inside RBCs appeared as dark-stained dots surrounded by pale areas in the cytoplasm. Furthermore, in infected neutrophils and monocytes, *A. phagocytophilum* and *Ehrlichia canis* multiplied organisms (morulae) appeared intracytoplasmic. *Anaplasma platys* appeared as basophilic inclusions in blood platelets. On the other hand, *Hepatozoon canis* gamont was not detected in peripheral blood smears (Fig. 2). Hemolymph smears of *R. sanguineus* (*s.l.*) examination revealed that *Babesia* spp. vermicules take many shapes, including round, amoeboid (3–4.8 µm), and club shaped with rounded and pointed ends, sized 6– 8 µm. Mature club-shaped *B. canis* vermicules appeared with an empty cytoplasm. Additionally, *H*. *canis* oocysts were described in hemolymph smears as ball-like structures packed with the dividing sporocysts; their size ranged from 150 to 200 µm. The light microscope could not detect sporozoites inside the oocyst. *Anaplasma* and *Ehrlichia* spp. were detected in hemolymph smears through some alterations in hemocytes. The plasmatocytes showed aggregates of them filling their cytoplasm (Fig. 3).

### Epidemiological data

The prevalence of TBPs among dogs was 23.56% (49/208), including *Anaplasma* and *Ehrlichia* with a prevalence of 11.1% (23/208), *Babesia canis* 8.2% (17/208), and mixed infections with two pathogens observed in 4.33% (9/208) of examined dog blood smears. However, *Hepatozoon canis* was undetected in blood smears (Table 3). Furthermore, TBP prevalence in hemolymph smears was 45.97% (251/546), including 35.89% (196/546) for *H*. *canis*, 8.1% (44/546) for *B. canis,* and 2.01% (11/546) for Anaplasmataceae*.*

**Table 3:** Prevalence of tick-borne pathogen infections related to some risk factors (season, age, sex, and breed) by microscopic examination of blood smears

Risk factor	Groups	Total examined dogs	*Babesia canis*		Anaplasmataceae		Mixed infection		Total	
			Infected dogs	%	Infected dogs	%	Infected dogs	%	Infected dogs	%
Season	Winter	62	3	4.84	4	6.45	1	1.61	8	12.9
	Spring	43	3	6.98	6	13.95	3	6.98	12	27.9
	Summer	47	6	12.77	0	21.28^a^	5	10.64	21	44.68^a^
	Autumn	56	5	8.93	3	5.36	0	0	8	14. 29
Age/year	< year	66	2	3.03	3	4.55	2	3.03	7	10.61
	1–5 years	98	11	11.22	16	16.33^a^	6	6.12	33	33.67^a^
	> 5 years	44	4	9.1	4	9.1	1	2.27	9	20.45
Sex	Male	135	10	7.41	14	10.37	6	4.44	30	22.22
	Female	73	7	9.59	9	12.33	3	4.11	19	26.02
Breeds	Long hair	111	8	7.21	10	9.01	6	5.41	24	21.62
	Short hair	97	9	9.28	13	13.4	3	3.1	31	25.77
Total		208	17	8.2	23	11.1	9	4.33	49	23.56

^a^Significant differences at *p* < 0.05, %: percentage of infection

**Fig. 4:**
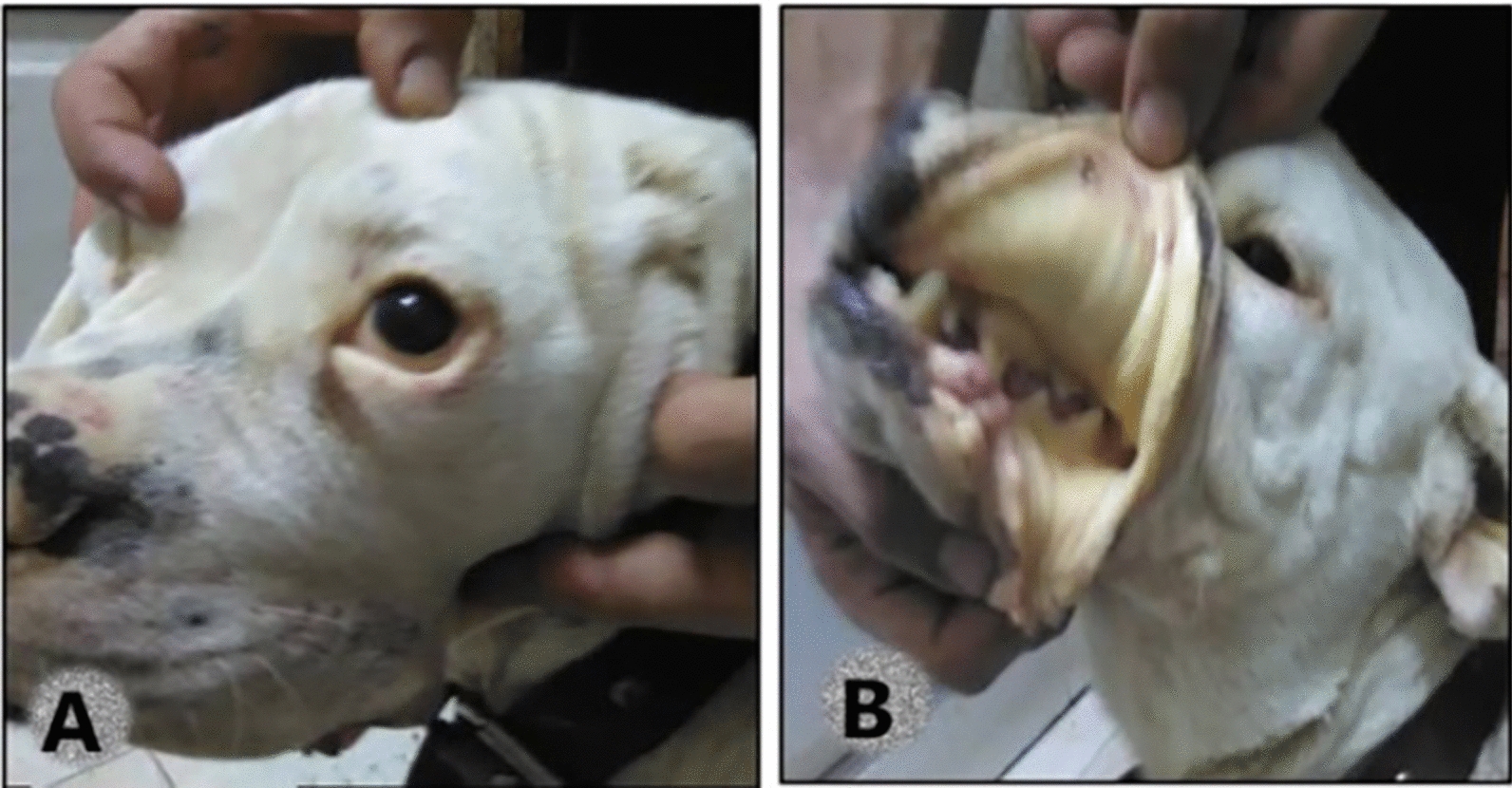
Signs of TBP infection in dogs (**A**): jaundice in sclera of eye (**B**): icteric mucosal membrane

Notably, clinical signs, including anorexia, depression, fever, lethargy, bleeding disorders, and jaundice or paleness of external mucosal membranes, were recorded in 31/49 infected animals, and the other 18 cases were healthy. Additionally, all infected animals were tick-infested (Fig. 4).

Regarding risk factor analysis from data obtained by microscopic examination, the chi-square (χ^2^) test revealed significant differences in season and age on the overall prevalence of TBPs in dogs, particularly for the Anaplasmataceae family (*P*-value < 0.05). At the same time, there was no significant difference between different breeds and sexes. Moreover, the *B. canis* infection rate was not significantly affected by variation in season, breeds, age, and sex (*P*-value > 0.05).

### Molecular findings

All blood samples that were positive on microscopic examination were also positive on PCR, and four samples from the negative samples on microscopic examination gave positive results by PCR. After PCR, the total prevalence rate of TBPs was 25.81% of the examined dog blood and 29.17% of the examined ticks. Anaplasmataceae family recorded the highest prevalence rate (19.35% and 20.83%) followed by *Babesia canis* (6.45% and 5.55%) in dog blood samples and ticks, respectively, while *H. canis* recorded the lowest prevalence rate (0% and 2.8%) in blood and ticks (Table 4). PCR amplified a monomorphic DNA fragment of 560-bp size of ssu-rRNA gene in case of *B. canis* and 670 bp of 18SrRNA gene in case of *H. canis*. The PCR product size of the Anaplasmataceae family was 450 bp of the 16SrRNA gene.

**Table 4:** Prevalence of tick-borne pathogen infection in dog serum samples and associated ticks by traditional and molecular techniques

Parasite	Total examined samples	*Babesia canis*		*Hepatozoon canis*		Anaplasmataceae		Total	
Method		Infected samples	%	Infected samples	%	Infected samples	%	Infected samples	%
Blood									
Blood smear	208	17	8.17	0	0	23	11.1	40	19.23
PCR	124	8	6.45	0	0	24	19.35	32	25.81
Tick									
Hemolymph smear	546	44	8.1	196	35.89	11	2.01	55	10.07
PCR	144	8	5.55	4	2.8	30	20.83	42	29.17

### Sequencing and phylogenetic analysis data

**Fig. 5:**
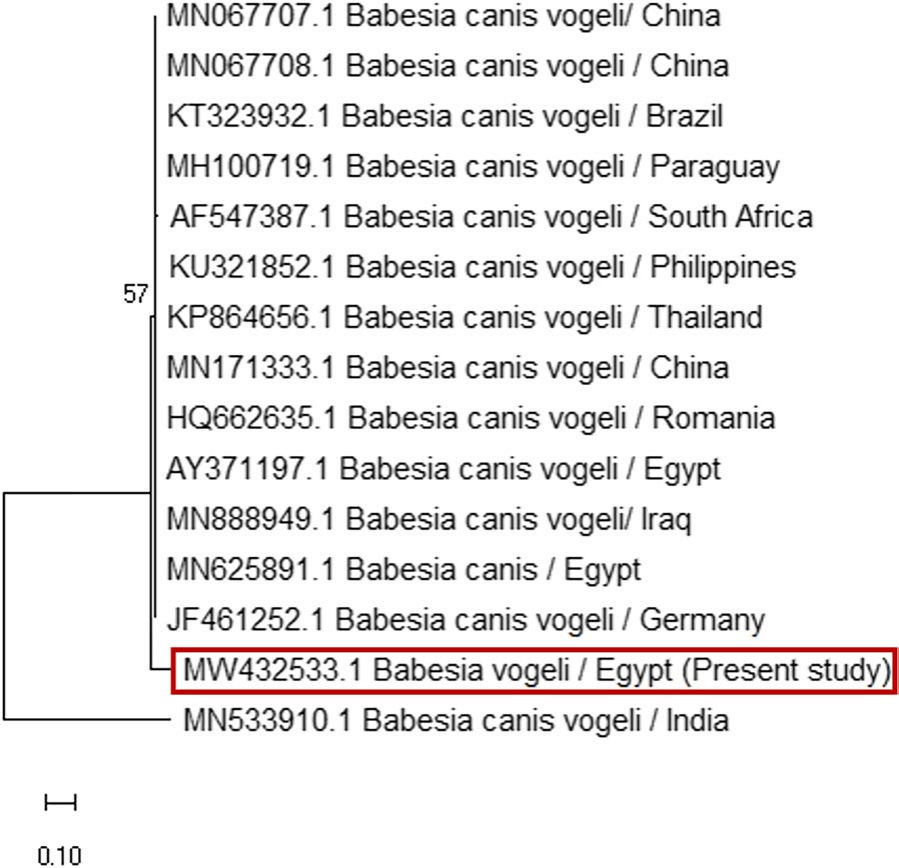
Phylogenetic relationships based on small subunit ribosomal RNA (ssu-rRNA) sequences of *Babesia canis vogeli*. The tree was constructed and analyzed using a maximum likelihood method and Tamura-Nei model, and numbers above internal nodes indicate the percentages of 1000 bootstrap replicates that supported the branch

**Fig. 6:**
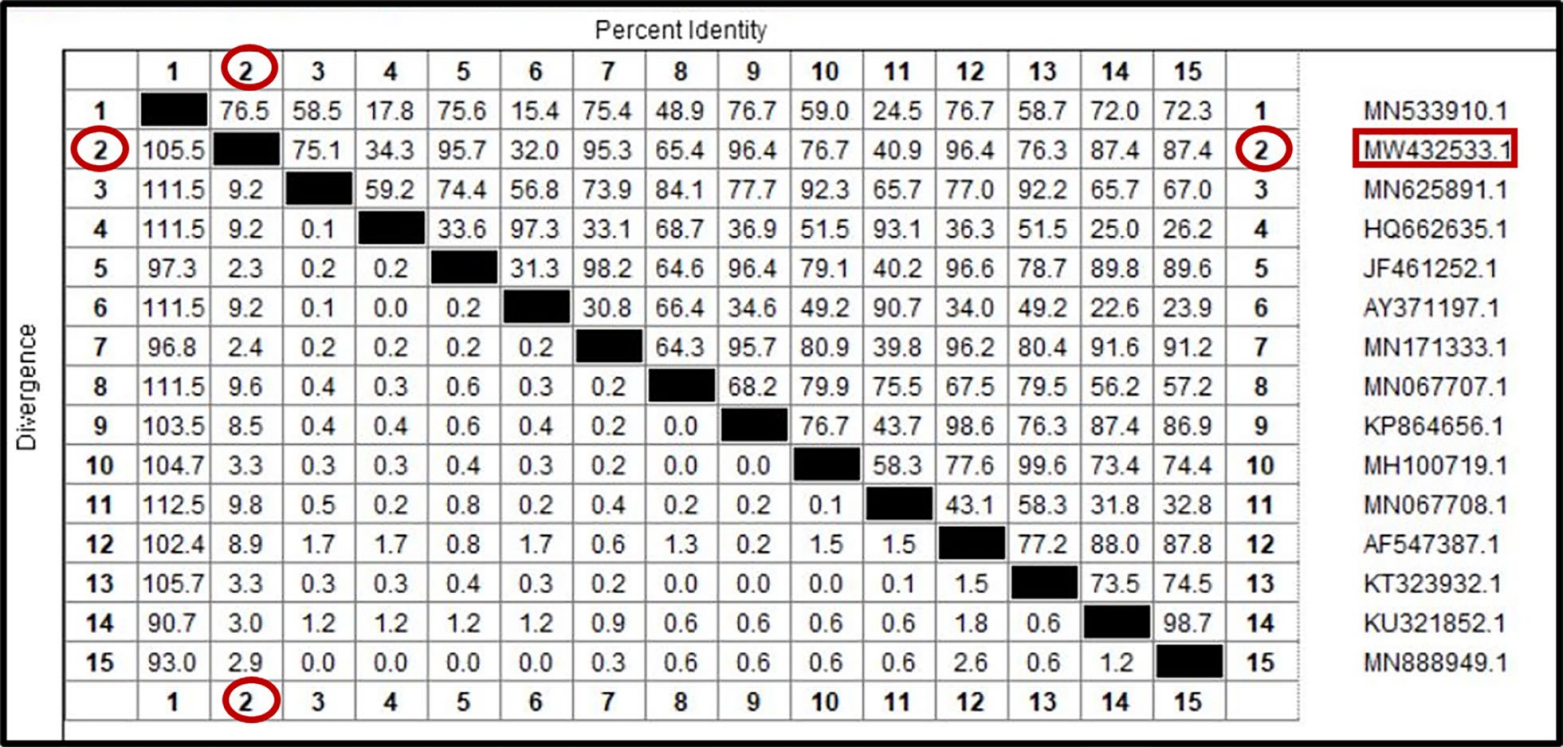
Similarity (percent identity) and genetic divergence of small subunit ribosomal RNA (ssu-rRNA) sequences of *Babesia canis vogeli* isolated from dog serum samples in Egypt (representing number, 2) compared with the most similar reference sequences (GenBank)

Sequence analysis identified six different species of tick-borne pathogens, namely *B. canis vogeli, H. canis, A. platys, A. phagocytophilum, A. marginale*, and *E. canis*. The data were accessed on GenBank. The phylogenetic analysis based on the ssu-rRNA sequences of *B. canis vogeli* isolated from dogs’ blood (MW432533) showed 96.4% similarity with *B. canis vogeli* from Thailand and South Africa (KP864656 and AF547387), respectively. However, the lowest genetic diversities between our isolate and other isolates on GenBank were 2.3% and 2.4%, with *B. canis vogeli* isolated from dogs in Germany and China (JF461252 and MN171333), respectively (Figs. 5, 6).

**Fig. 7:**
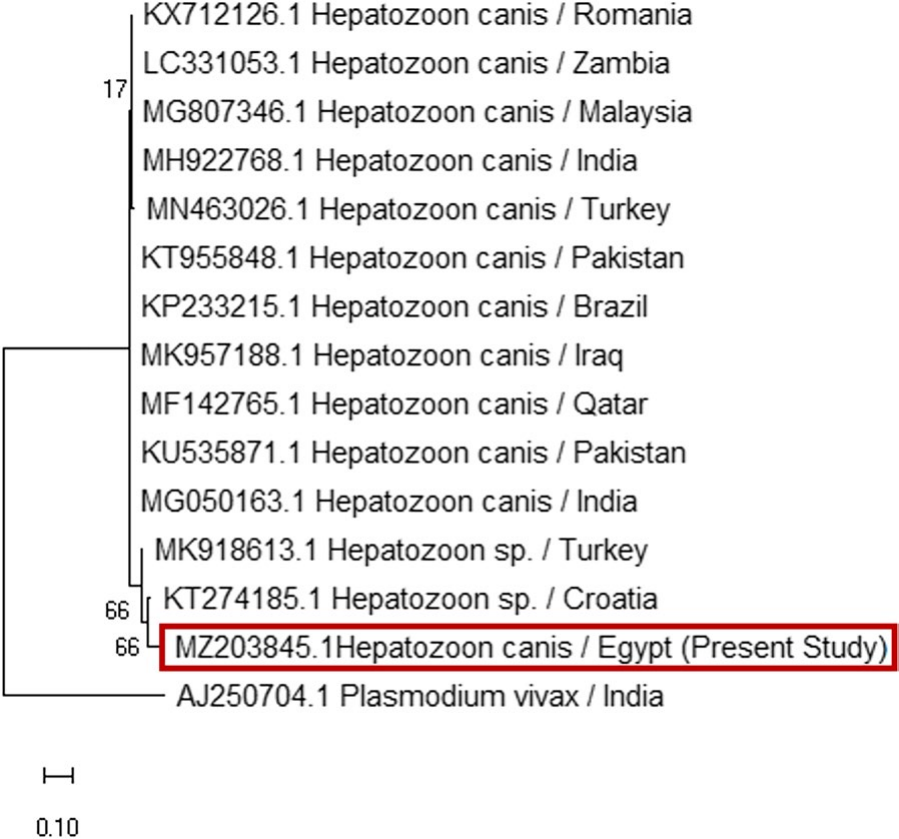
Phylogenetic relationships based on 18S ribosomal RNA (18S-rRNA) sequences of *Hepatozoon canis*. The tree was constructed and analyzed using a maximum likelihood method and Tamura-Nei model, and numbers above internal nodes indicate the percentages of 1000 bootstrap replicates that supported the branch

**Fig. 8:**
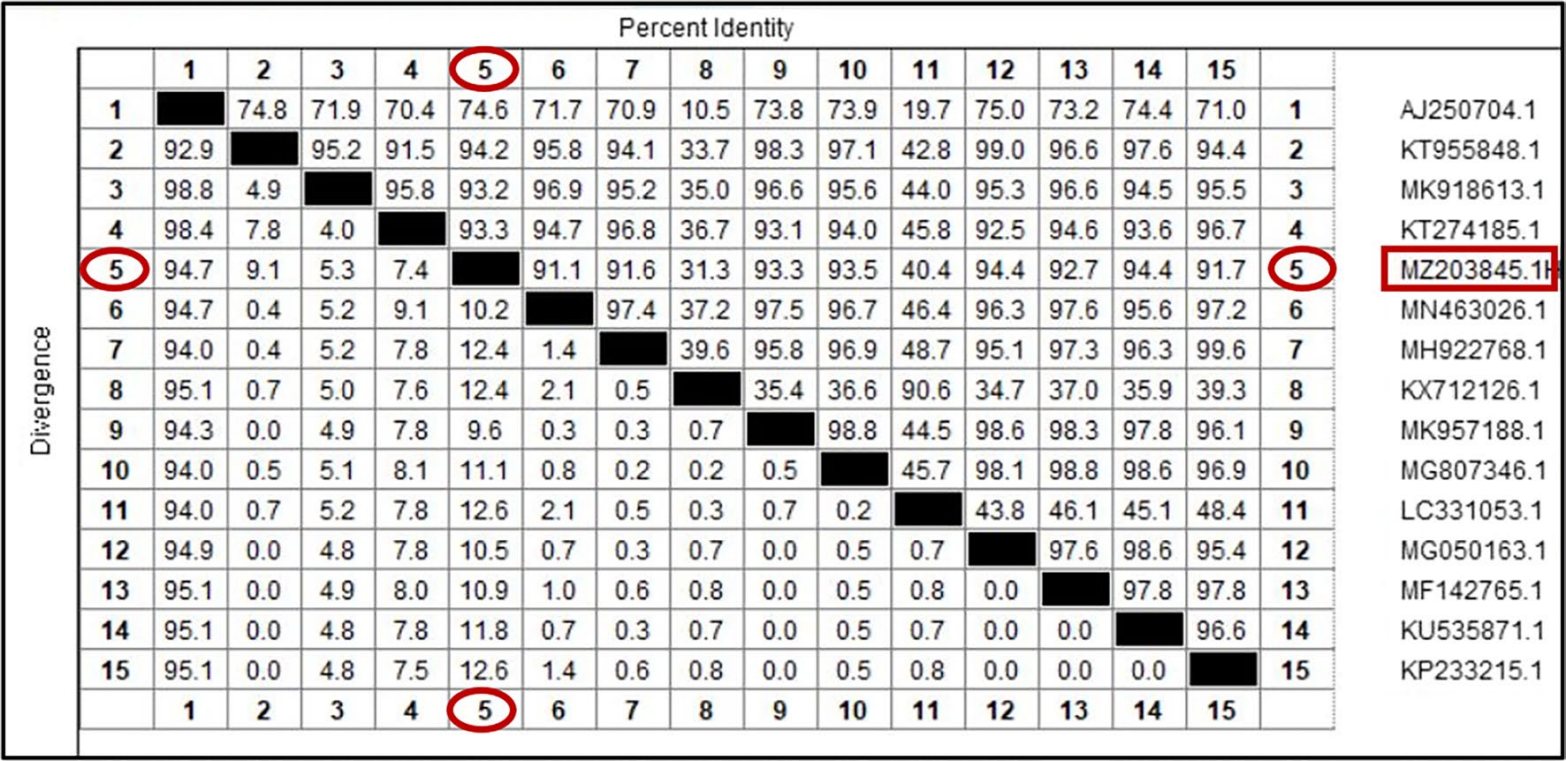
Similarity (percent identity) and genetic divergence of 18S ribosomal RNA (18S-rRNA) sequences of *Hepatozoon canis* isolated from *Rhipicephalus sanguineus* (s.l.) ticks collected from dogs in Egypt (representing number, 5) compared with the most similar reference sequences (GenBank)

The phylogenetic analysis of the current *H. canis* isolated from *R. sanguineus* (s.l.) (MZ203845) revealed a relatively high homology (94.4%) with *H. canis* isolated from Pakistan and India (KU535871 and MG050163). The lowest divergence ratios (9.1% and 9.6%) were detected with *H. canis* isolated from dog and red fox in Pakistan and Iraq (KT955848 and MK957188), respectively (Figs. 7, 8).

**Fig. 9:**
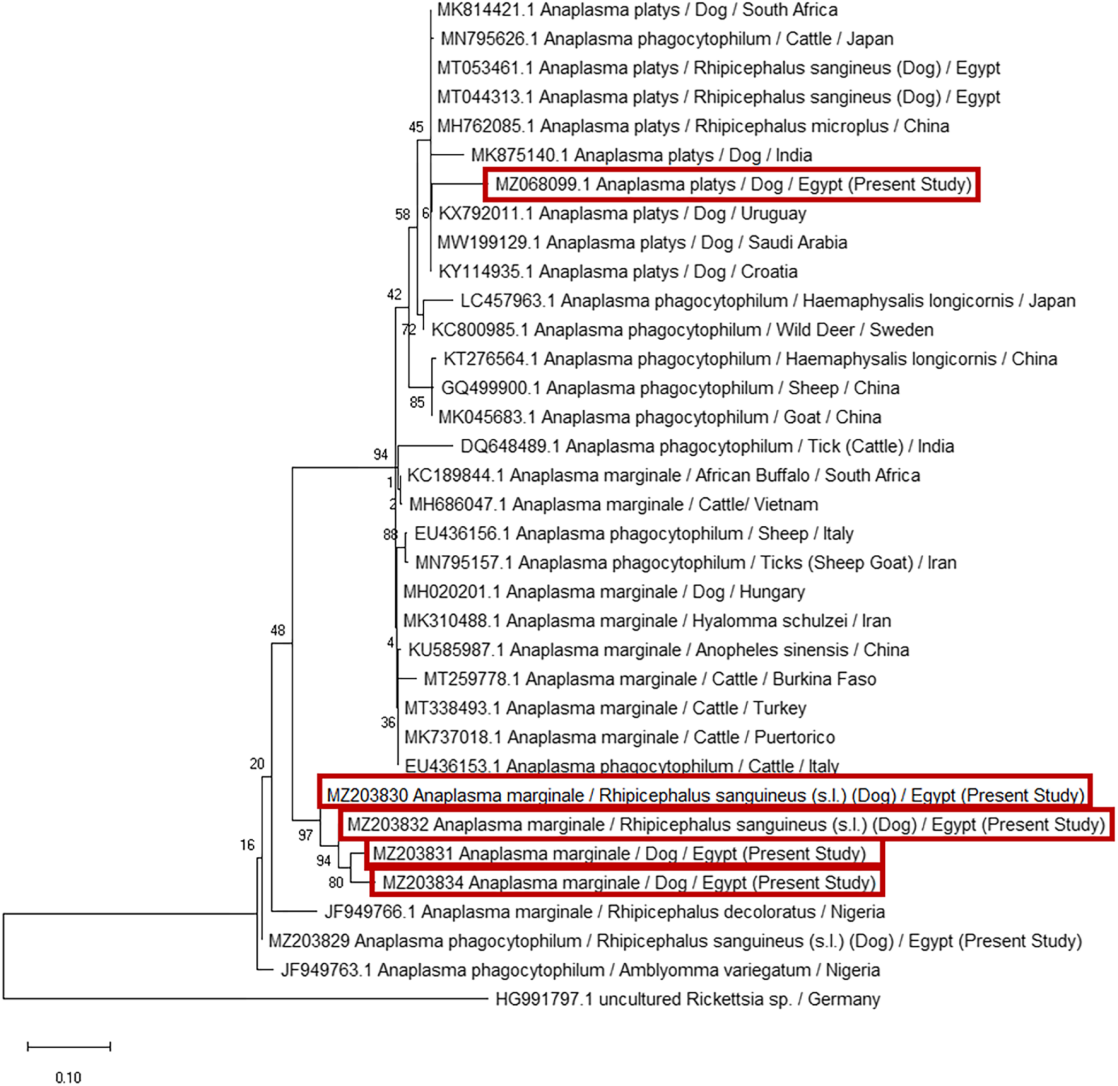
Phylogenetic relationships based on 16S ribosomal RNA (16S-rRNA) sequences of *Anaplasmataceae*. The trees were constructed and analyzed using a maximum likelihood method and Tamura-Nei model, and numbers above internal nodes indicate the percentages of 1000 bootstrap replicates that supported the branch

**Fig. 10:**
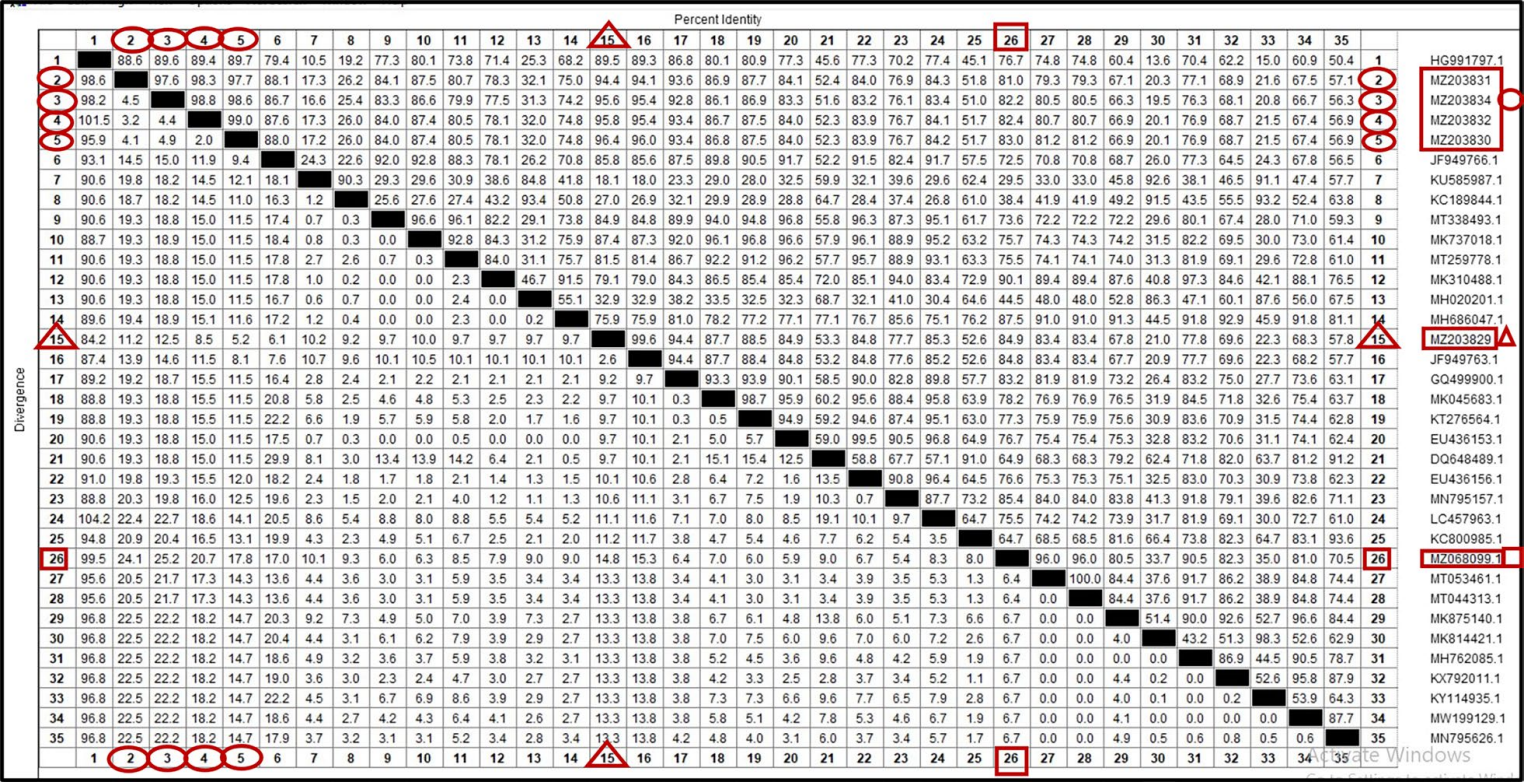
Similarity (percent identity) and genetic divergence of 16S ribosomal RNA (16S-rRNA) sequences of *A. platys, A. phagocytophilum*, and *A. marginale* isolated from dogs and *Rhipicephalus sanguineus* (s.l.) ticks in Egypt (representing number, 2–5, 15, 26) compared to the most similar reference sequences (GenBank). The 16S rRNA of *A. marginale* sequenced in this study is marked with an oval shape and represented as numbers 2–5, *A. phagocytophilum* is marked with a triangular shape and represented as number 15, and *A. platys* is marked with square shape and represented as number 26

The present study identified three different subspecies of *Anaplasma* (*A. platys, A. phagocytophilum*, and *A. marginale*) in dog blood and ticks. *Anaplasma platys* (MZ068099) was detected in dog, showing the highest percentage identity (96%) and the lowest divergence ratio (6.4%) with *A. platys* isolated from *R. sanguineus* (s.l.) in Egypt (MT053461). Additionally, *A. phagocytophilium* isolated from *R. sanguineus* (s.l.) showed high homology (99.6%) and low divergence ratio (2.6%) with *A. phagocytophilum* identified from *Amblyomma variegatum* collected from cattle in Nigeria (JF949763). Furthermore, the current study identified four sequences as *An*. *marginale* in dog and *R*. *sanguineus* (s.l.). When these sequences aligned, the highest genetic homology was obtained between *A. marginale* isolated from *R. sanguineus* (s.l.) (MZ203830 and MZ203832) with a similarity percentage (99%) and divergence ratio (2%), while the highest similarity between our *An*. *marginale* isolates and other isolates accessed on the GenBank was 91.24%, with *A. marginale* identified from *Rhipicephalus decoloratus* in Nigeria (JF949766) (Figs. 9, 10).

**Fig. 11:**
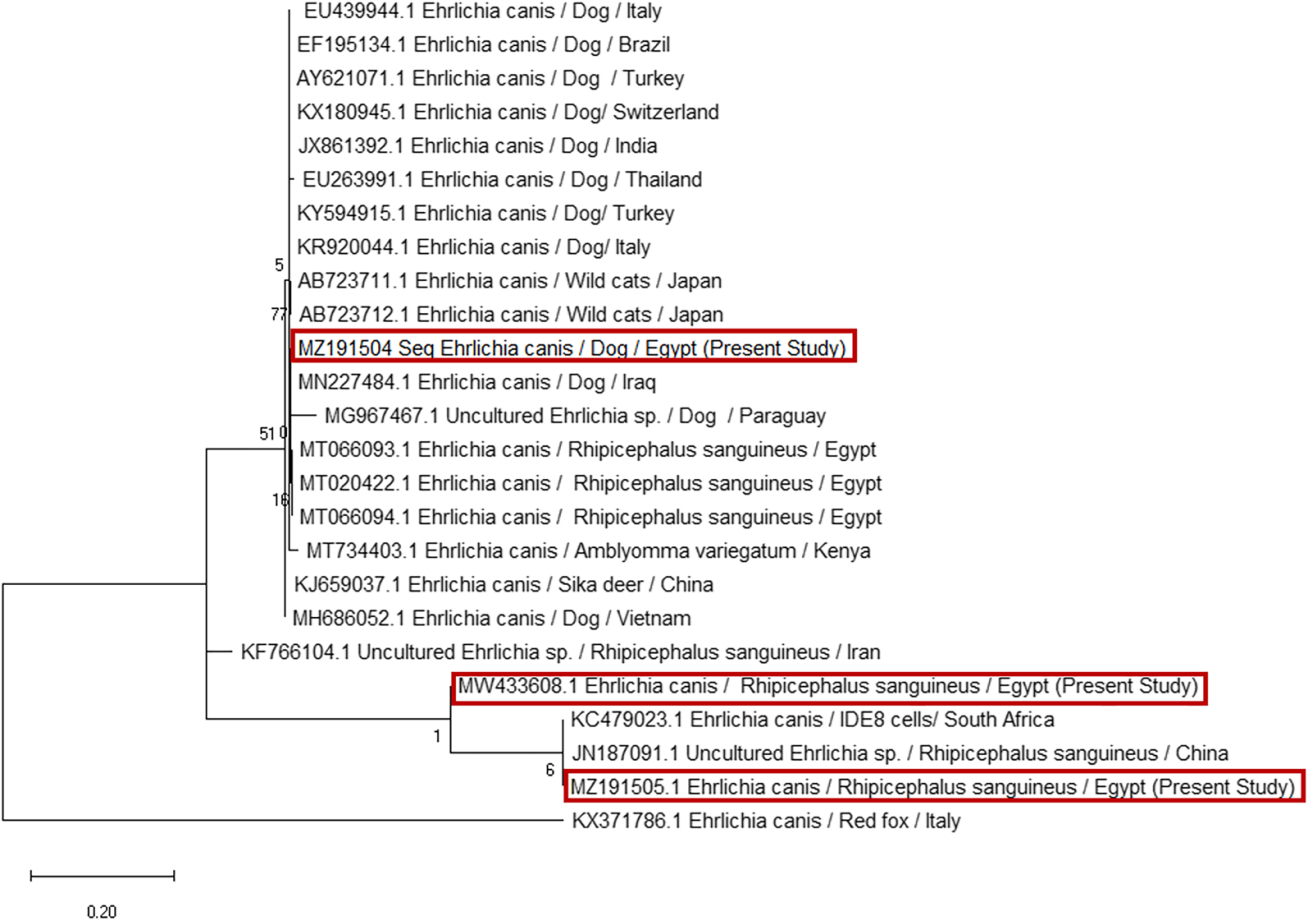
Phylogenetic relationships based on 16S ribosomal RNA (16S-rRNA) sequences of *Ehrlichia canis*. The trees were constructed and analyzed using a maximum likelihood method and Tamura-Nei model, and numbers above internal nodes indicate the percentages of 1000 bootstrap replicates that supported the branch

**Fig. 12:**
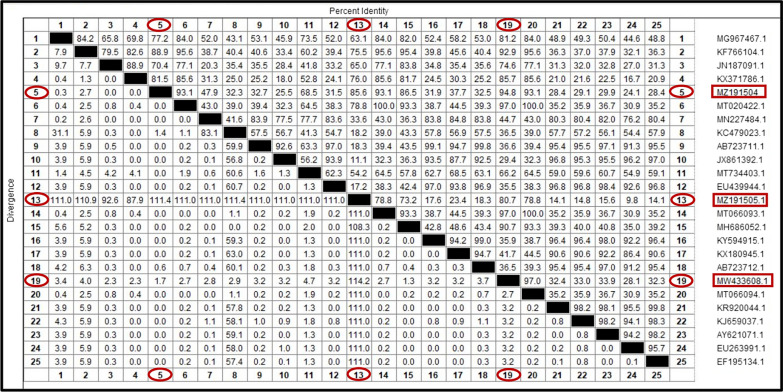
Similarity (percent identity) and genetic divergence of 16S ribosomal RNA (16S rRNA) sequences of *Ehrlichia canis* isolated from dogs and *Rhipicephalus sanguineus* (s.l.) ticks in Egypt (representing number 5, 13, 19) compared with the most similar reference sequences (GenBank). The 16S rRNA of *Ehrlichia canis* sequenced in this study is marked and represented as numbers 5, 13, 19

In the current phylogenetic analysis of *E*. *canis* isolates based on the 16S-rRNA sequences, three sequences of *E*. *canis* were isolated from dog and *R. sanguineus* (s.l.). After these sequences were aligned, a relatively high similarity (97%) with a very low genetic divergence ratio (2.7%) was recorded between *E. canis* isolated from *R. sanguineus* (s.l.) in Egypt (MW433608 and MT066094), respectively. Moreover, the current *E. canis* isolated from ticks (MZ191505) showed complete homology (100%) with *E. canis* isolated from *R. sanguineus* (s.l.) collected from dogs in Egypt (MT020422) (Figs. 11, 12).

Notably, the current isolates of *A. platys* isolated from dogs (MZ068099) had high similarity of 99.7% with *A. platys* isolated from humans in Mexico (MK386768).

## Discussion

The present study revealed that all TBP-infected cases were infested by *Rhipicephalus sanguineus* only. This observation agreed with previous reports in Egypt [24–26]. Clinical manifestations of TBDs mainly recorded in 63.27% (31/49) of infected animals, particularly *B. canis* infection, were detected in diseased dogs with fever, emaciation jaundice, and red urine. This was unlike what was observed by [27] who reported that canine babesiosis in Egypt usually takes the chronic form. The reason for our finding might be the weak immune status of infected dogs or the presence of mixed infections that increase disease expression.

Concerning the morphological description of the stages of *B. canis, H. canis, Anaplasma* spp., and *E. canis* in dog blood and tick hemolymph, they had the same morphology as reported previously [28–32]. The overall prevalence of TBPs by microscopic examination of blood smears in the present study was 23.56%. Among the positive cases, there was a higher prevalence of *Anaplasmataceae* family (*Anaplasma* spp. and *E. canis*) infection (11.1%) than *Babesia* (8.2%). These findings contrast previous records of [33] in India, who observed *Babesia* spp. infection in 88% of the positive cases with a higher prevalence than other blood parasites such as *E. canis* and *H. canis*. For *B. canis* the prevalence is nearly the same as that of previous studies [34, 35]; 6.7% and 7.47% prevalences were reported from naturally infected dogs from Tunisia and India, respectively. However, this is lower than what was detected by other findings [24, 26] reporting *Babesia* infection in 76.92% and 12.0% of examined dogs from different governorates in Egypt. *Anaplasma* spp. and *E. canis* prevalences were lower than that recorded in Malaysia (13.6%) [32] and higher than (4%) in Israel [36]. The current low prevalence might be because *Babesia* parasites are difficult to detect in blood smears except during the acute phase; thus, the negative results do not exclude the possibility of *Babesia* infection. Also, *E. canis* is difficult to detect by blood smear examination because of low parasitemia even during the acute stage of the disease [36]. This discrepancy in prevalence ratios could be attributable to the difference in sampling design and geographical variations.

Co-infections with two pathogens were observed microscopically in 9/208 (4.33%) of examined dogs. This result coincides with those of with many authors [37–39] who reported co-infection of *Ehrlichia/Anaplasma* with other pathogens in Brazil, Costa Rica, and the Philippines, respectively. This finding might be attributed to thrombocytopenia, usually found in dog cases infected with *Anaplasma platys* or *Ehrlichia canis* [40–42]. Another explanation for this finding is that vectors of these pathogens (*R. sanguineus*) (s.l.) can carry several pathogens simultaneously, which might lead to co-infection [43].

Regarding the prevalence rates of TBPs in hemolymph smears of *R. sanguineus, H. canis* was the most prevalent pathogen (35.89%), followed by *Babesia canis* (8.1%) and finally the Anaplasmataceae family (2.01%). *Hepatozoon* prevalence was lower than that recorded by [31] in Israel, who found that 85% of *R. sanguineus* (*s.l.*) that had artificially fed on a *Hepatozoon* infected dog were infected with *H. canis*. On the other hand, the current *B. canis* prevalence was much lower than that detected by [44] in Nigeria, who found 64.5% of hemolymph smears from five *Hyalomma* spp. detached from cattle. In addition, [45] reported a higher prevalence of *Anaplasma phagocytophilium* (51%) in hemolymph smears prepared from *Hyalomma* spp. collected from horses in Iraq. These variations in prevalence ratios might be due to differences in vector and vertebrate hosts. However, *H. canis* stages in hemolymph smears represented 35.89% not detected in blood smears. This finding is consistent with [46] who detected *H. americanum* oocysts in *A. maculatum* ticks collected from a dog that did not have any *H. americanum* stages in its blood or tissues. This result might be because oral intake of ticks is the main route of transmission of *Hepatozoon* spp. to dogs and not tick biting. Therefore, the possibility of dog infection from infected ticks is minimum. Another explanation is the long period elapsed (28 days) from oral tick intake until gamont appeared in the peripheral circulation. The infected adult ticks were still uninfective to dogs 40 days post molting [31, 47].

Concerning the epidemiological results, the overall seasonal prevalence of TBPs was most prevalent in warm seasons (summer 44.68% and spring 27.9%) compared to winters (12.9%). This result follows [33] in Chennai city, India, who found that blood parasite infections in dogs were more prevalent during the monsoon (windy) season. The probable reason behind this observation may be affected by the seasonal activity of the vector [*R. sanguineus* (s.l.)] in Egypt, which is maximum in summer. At the same time, complete disappearance occurs in the winter months, December and January [48]. Also, statistical analysis of risk factors detected a significant effect of age and no significant difference in different breeds and sex. This finding contrasts with [49] in Turkey, who found that the TBP infection rate was higher in adults than in young dogs. On the other hand, [33], in India no significant differences were found between different age groups, breeds, and sexes in the prevalence of canine vector-borne pathogens.

The effect of risk factors on *B. canis* infection rate showed no significant effects of age, sex, and breed. This finding parallels [27] and disagrees with [24, 50], who reported that canine babesiosis in Egypt infection increased by age; Malino and German Shepherds were more susceptible to infection. Furthermore, [51–53] in the US and Hungary recorded that some dog breeds such as Pit bull terriers, German shepherds, and heavy-coated Komondors were more susceptible to *Babesia* infection. In the Anaplasmataceae family, summer season and age significantly affected the infection rate, but no significant difference was recorded for sex and breeds. This result coincides with [8, 54, 55] in Egypt, Germany, and Morocco, while [56] reported that *Anaplasma* spp. and *Ehrlichia* spp. were more prevalent in female dogs and German shepherds in Egypt.

The total molecular prevalence of TBPs in dog blood was 25.81% including 6.45% for *Babesia canis* and 19.35% for the Anaplasmataceae family. The total prevalence was higher than [48, 57] 23.4% and 5.4% in Thailand and Turkey, respectively, and lower than [10, 58, 59] 72%, 45%, and 52% in Nigeria, Romania, and India, respectively. Regarding the present study, *B. canis* prevalence was nearly similar to (6%) [60] in Brazil and higher (0.6%, 1.9%, and 0.26%) than [61–63] in Nigeria, Palestine, and India, respectively. On the other hand, our finding was lower than [10, 50] 10.8% and 29.17% in dogs from Egypt and Romania. Concerning *Anaplasma* and *Ehrlichia*, the molecular prevalence was the same as that reported by [61], who recorded *Ehrlichia canis* in 12.7% and *A. platys* in 6.6% of examined dogs from Nigeria. While it was higher than what was detected by [49], *E. canis* was 4.9% and *A. platys* 0.5% in Turkey. Different prevalence worldwide might be due to variations in climatic conditions.

The overall molecular prevalence of TBPs in *R. sanguineus* (s.l.) was 29.17%, including 20.83% for the Anaplasmataceae family, followed by 5.55% for *Babesia canis* and 2.8% for *H. canis*. The highest prevalence of *B. canis vogeli* (8.7%) was recorded by [60] in *R. sanguineus* (s.l.) collected from dogs in Brazil. On the other hand, a lower prevalence (0.5%) was detected by [62] in *R. sanguineus* (s.l.) collected from dogs in Palestine. In *Anaplasma* and *Ehrlichia* spp. infection rates, our findings are higher than [64, 65] who detected *A. phagocytophilum, A. platys*, and *E. canis* in *R. sanguineus* (s.l.) ticks collected from dogs in Egypt with ratios of 13.7%, 1.32%, and 1.98%, respectively, while higher prevalence rates for *Anaplasma* and *Ehrlichia* spp. of 21.1% and 45.5% were reported by [61, 66] in Nigeria and Iran, respectively. The present low ratio of *Babesia* spp. and *H*. *canis* in infected ticks might be caused by many PCR inhibitors in the extracted DNA from ticks.

Regarding the sequencing and phylogenetic analysis, the present study genetically identified six tick-borne pathogens in dog blood samples and *R. sanguineus* (s.l.), including *B. canis vogeli, H. canis, A. platys, A. phagocytophilum, A. marginale*, and *E. canis*. The current study found that *B. canis vogeli* was the only species responsible for canine babesiosis in Egypt. This finding agreed with [24, 27, 50]. Moreover, *A. marginale* and *A. phagocytophilum* identified in the current study from tick [*R. sanguineus* (s.l.)] showed relatively high homology (96.4%) with each other. This result is consistent with the observation of [67] in Spain, who mentioned that *A. marginale* and *A. phagocytophilum* shared similarities at a molecular level. *Anaplasma phagocytophilum* detected in *R. sanguineus* (s.l.) shared high similarity (99.6%) with *A. phagocytophilum* detected in *Amblyomma variegatum* attached to cattle in Nigeria [68]. Notably, this is the first report of DNA of *A. marginale* in dogs and their associated *R. sanguineus* (s.l.) ticks in Egypt. *Anaplasma marginale* was also detected in dogs from Hungary and registered in the GenBank under accession number (MH020201) in an unpublished report. This finding supported that some TBPs accidentally parasitized other hosts, as reported by [69].

*Ehrlichia canis* showed complete homology (100%) with *E. canis* isolates from *R. sanguineus* (*s.l.*) collected from dogs in Egypt [65]. Furthermore, the current isolates of *A. platys* isolated from dog serum samples and ticks showed high similarity (99.7%) with *A. platys* isolated from humans in Mexico. This observation is alarming because of the zoonotic potentiality of these species, similar to a previous observation in Egypt [65].

## Conclusion

The present study detected a wide range of TBPs (*B. canis, H. canis, A. platys, A. phagocytophilum, A. marginale*, and *E. canis*) that indicates the emergence of tick-borne diseases in domestic animals and humans in Egypt. However, these diseases might be misdiagnosed because of the lack of public health and veterinary resources, increasing the possibility of human infection. *Anaplasma marginale* is a non-canine tick-borne pathogen identified in dogs as an accidental host. One of the most important findings is the high genetic similarity percentage between the detected *E. canis* and *A. platys* isolates with other species isolated from humans in Egypt and other countries. This observation is alarming because of the zoonotic potentiality of these species. To the best of our knowledge, this is the first time that *H. canis* and *A. marginale* were detected in dogs and associated ticks in Egypt.

## Data Availability

The datasets generated or analyzed during the current study are available in the Gene Bank repository, accsession numbers: MZ191504, MZ068099, MZ203831, MZ203834, MW432533, MW433608, MZ203829, MZ203832, MZ203845 and MZ203830.
